# Use of a smartphone app to inform healthcare workers of hospital policy during a pandemic such as COVID-19: A mixed methods observational study

**DOI:** 10.1371/journal.pone.0262105

**Published:** 2022-01-05

**Authors:** R. I. Helou, C. M. Waltmans–den Breejen, J. A. Severin, M. E. J. L. Hulscher, A. Verbon

**Affiliations:** 1 Department of Medical Microbiology and Infectious Diseases, Erasmus Medical Center, Rotterdam, The Netherlands; 2 Scientific Center for Quality of Healthcare (IQ healthcare), Radboud Institute for Health Sciences, Radboud University Medical Center, Nijmegan, The Netherlands; University of Maryland School of Medicine, UNITED STATES

## Abstract

**Objective:**

To evaluate the use of a COVID-19 app containing relevant information for healthcare workers (HCWs) in hospitals and to determine user experience.

**Methods:**

A smartphone app (Firstline) was adapted to exclusively contain local COVID-19 policy documents and treatment protocols. This COVID-19 app was offered to all HCWs of a 900-bed tertiary care hospital. App use was evaluated with user analytics and user experience in an online questionnaire.

**Results:**

A total number of 1168 HCWs subscribed to the COVID-19 app which was used 3903 times with an average of 1 minute and 20 seconds per session during a three-month period. The number of active users peaked in April 2020 with 1017 users. Users included medical specialists (22.3%), residents (16.5%), nurses (22.2%), management (6.2%) and other (26.5%). Information for HCWs such as when to test for SARS-CoV-2 (1214), latest updates (1181), the COVID-19 telephone list (418) and the SARS-CoV-2 / COVID-19 guideline (280) were the most frequently accessed advice. Seventy-one users with a mean age of 46.1 years from 19 different departments completed the questionnaire. Respondents considered the COVID-19 app clear (54/59; 92%), easy-to-use (46/55; 84%), fast (46/52; 88%), useful (52/56; 93%), and had faith in the information (58/70; 83%). The COVID-19 app was used to quickly look up something (43/68; 63%), when no computer was available (15/68; 22%), look up / dial COVID-related phone numbers (15/68; 22%) or when walking from A to B (11/68; 16%). Few respondents felt app use cost time (5/68; 7%).

**Conclusions:**

Our COVID-19 app proved to be a relatively simple yet innovative tool that was used by HCWs from all disciplines involved in taking care of COVID-19 patients. The up-to-date app was used for different topics and had high user satisfaction amongst questionnaire respondents. An app with local hospital policy could be an invaluable tool during a pandemic.

## Introduction

In the first months of the COVID-19 pandemic, and even more thereafter, an overwhelming number of publications appeared on COVID-19. Especially in the first months, clinically relevant new findings were published on a daily basis while healthcare workers (HCWs) and hospital management struggled with an enormous influx of COVID-19 patients in the hospitals. Guidelines which assist HCWs in clinical decision making have shown to have a beneficial effect on therapy, length of hospital stay, mortality and overall patient safety [1, 2]. However, for COVID-19, these had to be changed regularly and often on a daily basis. Since clear, centrally directed communication during a healthcare crisis is key, hospitals should ensure correct and current policy is disseminated in a concise manner to all HCWs [3, 4]. HCWs have to be able to rely on the latest information provided, thereby ensuring good quality outbreak response.

Information technology such as clinical decision support systems (CDSS) can positively impact healthcare by facilitating diagnostic [5], therapeutic [6] and prognostic [7] processes. CDSS is software that facilitates clinical decision making, either by comparing individual patient characteristics with a clinical knowledge database or by providing relevant information which is then combined with the knowledge of the clinician [8]. Such systems also have shown value in the COVID-19 outbreak management setting [9–11]. A multitude of smartphone applications (apps) for patients have been developed and evaluated during the COVID-19 pandemic with focus on contact tracing [12, 13], self-testing [14], self-reporting [15] and education [16]. While these apps can help inform the public, professionals, and policy-makers, they are dependent on user uptake and continued engagement. In addition, ethical issues must be addressed to enable professionals and policy-makers to effectively use the data collected while protecting the privacy of app users. Few apps specifically for HCWs, some including CDSS, have been studied and these were limited to instruction videos [17], policy documents [18] or the addition of COVID-19 content to an existing platform containing an array of clinical medical content [19]. Although apps may have high potential to distribute the latest vital hospital information and policy on COVID-19 amongst HCWs, few studies evaluated the use of such apps focusing solely on COVID-19. The aim of this study is to evaluate the use of a COVID-19 app containing relevant information (such as local infection prevention guidelines, clinical guidelines, instructions on the use of personal protective equipment (PPE) and frequently asked questions) for HCWs in hospitals and to determine user experience.

## Methods

### Setting

This study was conducted in the Erasmus MC University Medical Center (Erasmus MC), Rotterdam, The Netherlands, an academic, tertiary care hospital with approximately 900 beds and 14,359 HCWs including 2,512 nurses and 935 medical specialists. The overall number of COVID-19 admissions in the hospital was monitored by the central hospital administration. Updates in local policy of the Erasmus MC were published on the intranet of the hospital, and communicated via the heads of the departments.

### Eligibility criteria

All HCWs of the hospital with an institutional e-mail address were eligible to use the app.

### Intervention

#### App

The clinical decision support platform Firstline [20], was adapted to local conditions with exclusively SARS-CoV-2 / COVID-19 content from the Erasmus MC: the COVID-19 app, using Firstline’s cloud-based content management system (CMS). The mobile COVID-19 app was available for iOS and Android. In order to avoid inappropriate off-site usage of locally-customized guidelines, potential users of the mobile COVID-19 app were required to authenticate using their institutional email address.

#### Content

Content included local infection prevention guidelines, clinical guidelines (diagnostic and therapeutic, including drug monographs), instructions on the use of personal protective equipment (PPE) such as face masks, goggles, face shields and medical gowns, COVID-19 related phone numbers of the hospital which could be dialed instantly and frequently asked questions. All content was non-confidential and available to all HCWs. App updates coincided with updates in infection prevention and clinical guidelines, which sometimes took place on a daily basis. Updates were created and reviewed in a restricted environment and were only released to end users upon approval. Therefore, updates in the app may have been published several minutes later than the content on the hospital’s intranet. Additionally, updates were most frequently uploaded on the intranet and in the app in the evening. To ensure that users could easily access the latest updates, the top page of the COVID-19 app -named ‘latest updates’- was dedicated for this purpose with a chronological overview.

#### Recruitment

A newsflash and an article about the COVID-19 app and its purpose was published on the intranet of the hospital on March 31, 2020. After one day on the front page the article moved down in the chronological newsfeed. A link in the article redirected potential app users either to iTunes (Apple) or the Play store (Google). Five weeks after app launch, a second article was published on the intranet informing HCWs about the app and its use in the first month (May 6, 2020). Six weeks after app launch, users were asked to fill out a questionnaire to evaluate their user experience with the app in a sticky message on the app home screen with a link to the online questionnaire. This message was shown until the end of the study on July 8, 2020. Simultaneously, a push message was sent to all app users informing them about the questionnaire study and asking for their participation. Additional e-mails were sent to app user groups (based on role) with a relatively low response rate during preliminary analysis.

#### Outcomes

Primary outcomes were 1) process indicators -including number of app downloads, session time, and accessed pages- which were anonymously collected and made available on a secure dashboard by Firstline and 2) user experience, focusing on barriers and facilitators of app use and use in general during the COVID-19 pandemic.

#### Questionnaire

User experience was evaluated in an online questionnaire using LimeSurvey v2.06, an open-source online survey software. The questionnaire contained 23 questions and was developed by the authors based on interviews from a pilot study with an antibiotic stewardship app [21] and available literature [22–25]. In this pilot study 18 purposefully sampled potential users from several departments in three different countries (Sweden, Switzerland and The Netherlands) were interviewed. After two weeks of app use each user was interviewed about their experiences. Additional users were interviewed until information saturation was reached. The interview questions were based on the Flottorp framework, which is designed to evaluate barriers and facilitators of an intervention [22]. For analysis of the interviews the software Atlas.ti was used. Based on the Flottorp framework, the findings of the interviews and additional literature, the current survey was developed. The preliminary data has not yet been published as it is part of the abovementioned trial. Questions of the COVID-19 app survey focused on content and app use in general, especially during a busy period caused by SARS-CoV-2 / COVID-19 (Tables 3, 4 and 5, S1 Appendix).

### Data management and analysis

During the installation process of the app, users were informed that user analytics would be collected and anonymously analyzed on group level. Additionally, users were asked to select their role in the hospital. Data of process indicators was stored in Data Studio, Google. Responses on the questionnaire were stored in LimeSurvey which was hosted on secure, password-protected hospital servers. In the questionnaire basic demographic information such as age, gender, role and department was asked. Data obtained from the questionnaire was collected and analyzed anonymously. Descriptive analyzes and Fisher’s exact test to assess questionnaire responses association were performed using IBM SPSS Statistics 25.0 for Windows (IBM, Armonk, NY, USA).

### Ethics and consent

Ethical approval for this study was obtained from the institutional review board of the Erasmus MC, Rotterdam, The Netherlands, number MEC-2020-0406. All data was anonymized.

## Results

### COVID-19 admissions

The first COVID-19 patient was admitted to our hospital at the end of February 2020. After a peak of admissions in (mainly the second half of) March 2020 with 135 patients with a positive SARS-CoV-2 PCR, the number of patients with a positive SARS-CoV-2 PCR declined in April (111), May (20), June (5) until July 8 (1). In total, 272 patients with a positive SARS-CoV-2 PCR were admitted until July 8, 2020. Similarly, on a national level COVID-19 admissions in February 2020 were few (9), peaked in March 2020 (6373) and decreased in the following months (4845 in April, 904 in May, 205 in June and 166 in July) [26].

### User analytics

**Fig 1:**
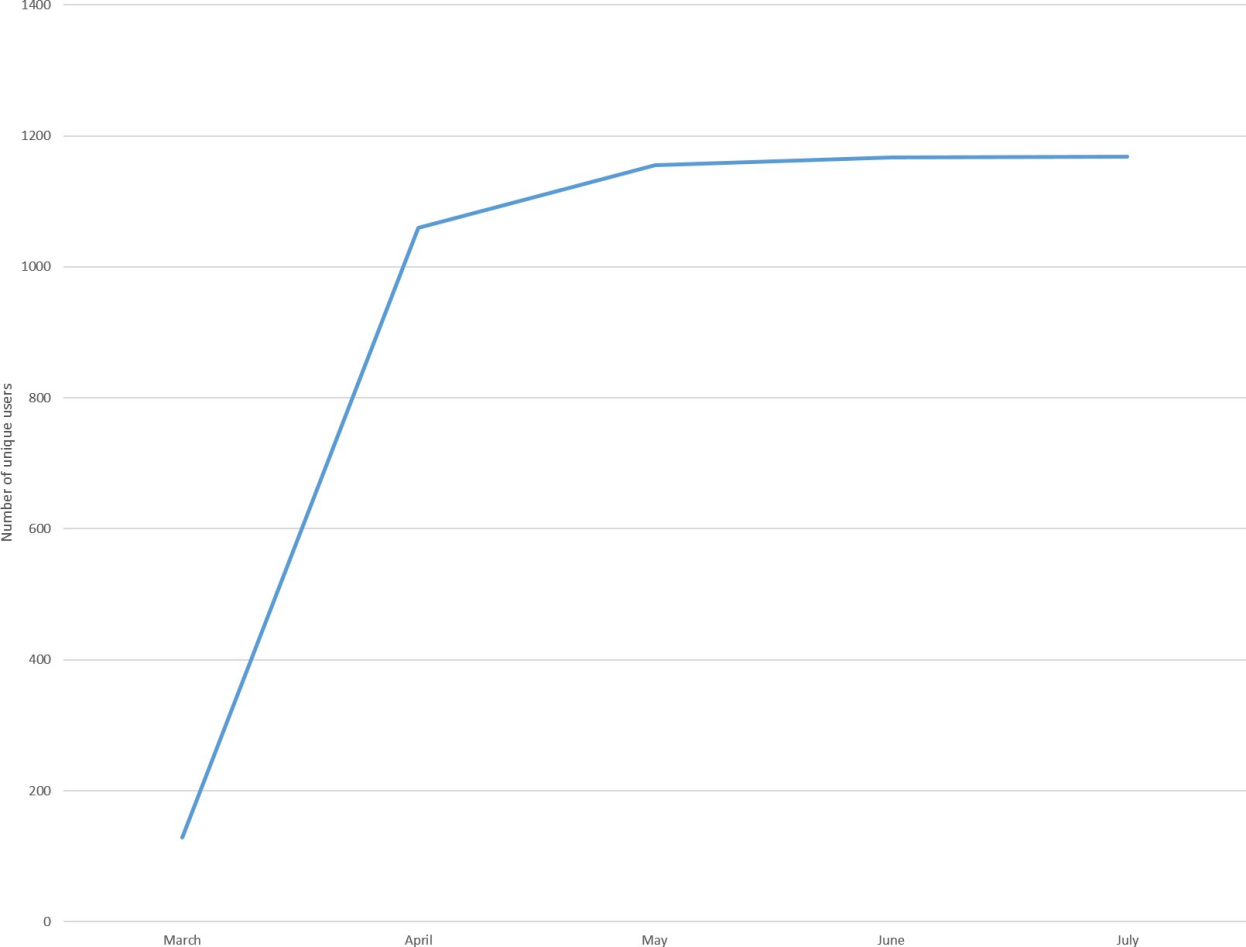
Monthly adoption rate of COVID-19 app from March 30, 2020 to July 8, 2020.

**Fig 2:**
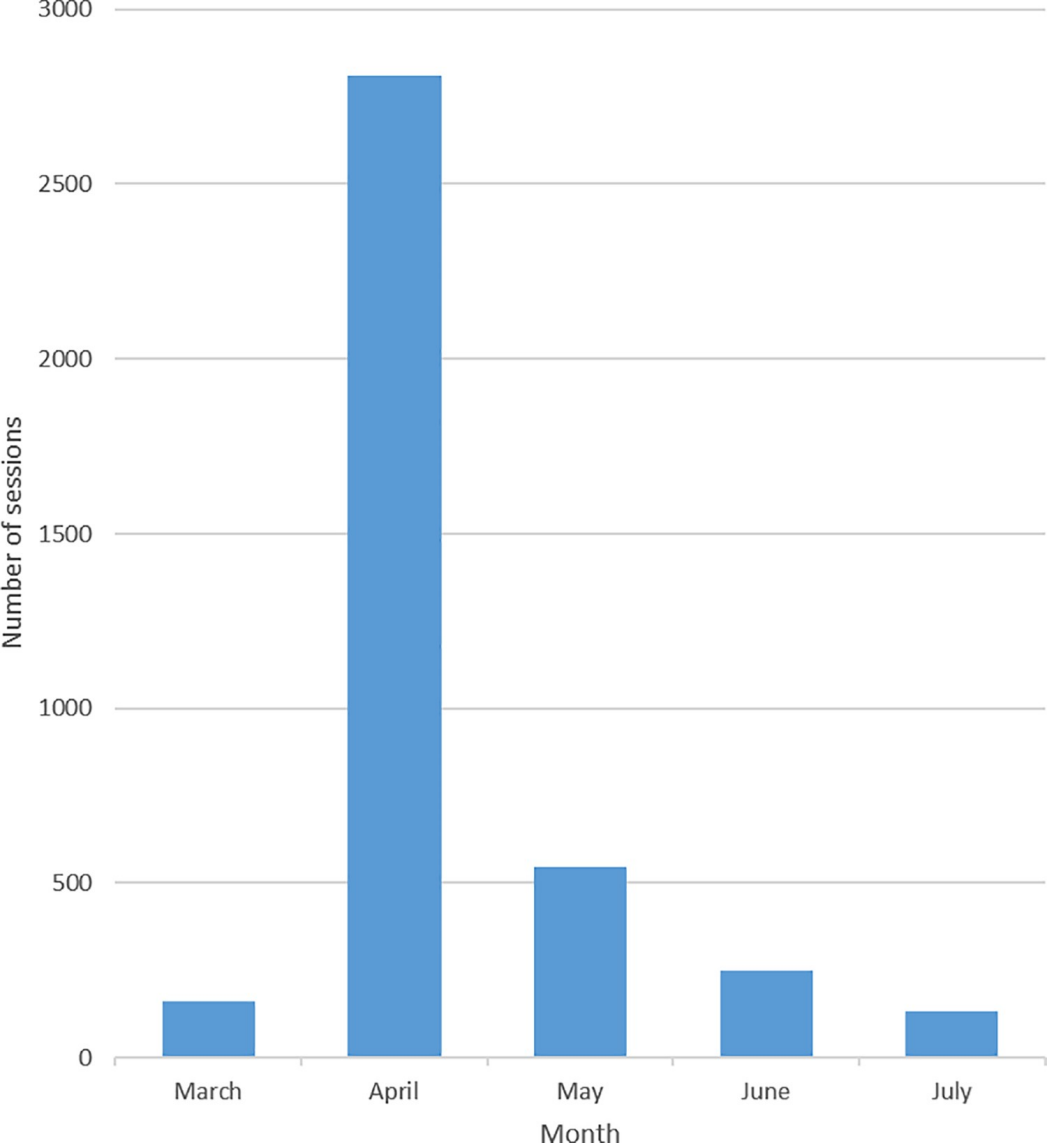
Number of COVID-19 app sessions per month.

The COVID-19 app was introduced on March 30, 2020. From the introduction until the end of study app content was updated 156 times. The total number of users from March 30, 2020 to July 8, 2020 was 1,168 of the hospital’s 14,359 HCWs (8.1%) which included 27.9% of all medical specialists and 10.3% of all nurses. COVID-19 app users were predominantly in the role of medical specialist (22.3%), nurse (22.2%), resident (16.5%), management (6.2%), medical student (4.0%), fellow (2.1%), infection prevention expert (0.4%) and other (26.5%) (Fig 1, S2 Appendix). The group ‘other’ consisted primarily of technicians (such as radiologic, laboratory, operating room, medical, IT, anesthetic, maintenance and pharmacy technicians) (38.4%), administrative professionals (30.8%), clinicians (16.1%), and research and education professionals (9.0%). The number of active users varied during the study period from 128 (March), 1017 (April), 594 (May), 487 (June) to 368 (July). The total number of sessions was 3903 with peak accessions at nine o’clock in the morning (Fig 2, S1 Fig). Nurses had the highest number of sessions throughout the day (Table 1).

**Table 1:** Number of sessions per role per time of day.

8:00 AM– 5:00 PM	5:00 PM– 11:00 PM	11:00 PM– 8:00 AM
Nurse (418)	Nurse (222)	Nurse (99)
Other (345)	Other (205)	Medical specialist (63)
Medical specialist (332)	Medical specialist (138)	Other (54)
Resident (287)	Resident (128)	Resident (40)
Management (92)	Management (26)	Management (13)
Medical student (38)	Medical student (15)	Fellow (5)
Fellow (35)	Physician assistant (7)	Medical student (5)
Physician assistant (35)	Fellow (6)	Physician assistant (4)
Infection prevention expert (3)	Infection prevention expert (2)	Infection prevention expert (1)

The average session time was 1 minute and 20 seconds and most users used the app for 2–4 sessions during the study period (Table 2).

**Table 2:** Sessions per role.

2–4 sessions	5–10 sessions	>10 sessions
Other (130)	Nurse (58)	Nurse (16)
Medical specialist (108)	Other (45)	Other (9)
Nurse (104)	Medical specialist (39)	Medical specialist (8)
Resident (75)	Resident (21)	Resident (4)
Management (26)	Management (10)	Management (3)
Medical student (24)	Fellow (5)	Fellow (1)
Physician assistant (8)	Physician assistant (4)	Infection prevention expert (0)
Fellow (6)	Medical student (3)	Medical student (0)
Infection prevention expert (1)	Infection prevention expert (1)	Physician assistant (0)

Overall, the most frequently accessed advice was information for HCWs such as when to test for SARS-CoV-2 (1214), latest updates (1181) and the COVID-19 telephone list (418). An advice can contain several screens with information. The most accessed screens were HCWs with possible COVID-19 symptoms (596), no / unknown unprotected physical contact with COVID-19 patients (472) and HCW without fever (461) (S3 Appendix). Among nurses, medical specialists and residents the most frequently accessed advice was information for HCWs such as when to test for SARS-CoV-2, latest updates and information regarding patients.

### User experience

The initial app push message to invite users to respond to the questionnaire yielded 11 respondents. Three subsequent e-mails were sent as a reminder and yielded an additional 24, 22 and 14 respondents, respectively.

Of the COVID-19 app users, 71 users with a mean age of 46.1 years from 19 different departments completed the questionnaire of which three users partially completed the questionnaire. Most respondents were female (53/71; 75%), and nearly all respondents agreed that use of smartphone apps can contribute to their work (68/71; 96%) and indicated to already use different apps for their work on a daily (21/71; 30%) or weekly (18/71; 25%) basis (Table 3).

**Table 3:** Characteristics of questionnaire respondents (N = 71).

Age group, n (%)		Value
	20–29	10 (14)
	30–39	9 (13)
	40–49	18 (25)
	50–59	28 (39)
	60–69	6 (9)
**Gender, n (%)**		
	Female	53 (75)
	Male	18 (25)
**Role, n (%)**		
	Management	10 (14)
	Medical specialist	13 (18)
	Nurse	22 (31)
	Other	19 (27)
	Physician assistant	3 (4)
	Resident	4 (6)
**Number of departments**		19
**Thoughts on smartphone app use for work, n (%)**		
	Apps can contribute to my work	68 (96)
	Apps don’t contribute to my work	1 (1)
	No opinion	2 (3)
**Frequency of smartphone app use for work, n (%)**		
	A few times a month	18 (25)
	A few times a week	18 (25)
	Every day	21 (30)
	I don’t use smartphone apps for work	14 (20)
**Familiarity with advice in COVID-19 app** ^**a**^ **, n (%)**		
	Familiar	18 (26)
	Partially familiar	49 (72)
	The advice was new to me	1 (1)

^a^Data missing for familiarity with advice, (n = 3).

Questionnaire respondents considered the COVID-19 app clear, easy-to-use, fast, and useful (Table 4). One percent (1/70) of the respondents did not trust the app.

**Table 4:** Impressions of the COVID-19 app by questionnaire respondents (N = 70).

Clear, n (%)		Value
	Yes	54 (92)
	No	5 (8)
**Easy-to-use, n (%)**		
	Yes	46 (84)
	No	9 (16)
**Fast, n (%)**		
	Yes	46 (88)
	No	6 (12)
**Trustworthy, n (%)**		
	Yes	45 (64)
	Yes, after I checked it on accuracy	13 (19)
	A little	11 (16)
	No	1 (1)
**Useful, n (%)**		
	Yes	52 (93)
	No	4 (7)

The most frequent reasons to use the app were checking the latest SARS-CoV-2 / COVID-19 updates, infection prevention and control policy, looking up/dial telephone numbers and clinical advice. Sixty-seven users were already partially familiar (49/68; 72%) or familiar (18/68; 26%) with the COVID-19 app content. Hyperlinks to the source documents were available, especially if the information was too elaborate to show in the app. Sixty-eight users rated the presence of hyperlinks as neutral (30/68; 44%), annoying (25/68; 37%) or did not encounter the hyperlinks (13/68; 19%). The most stated reasons to use the COVID-19 app were a quick information check and while ‘on the go’, such as when no computer was available. Furthermore, 63% (43/68) of the users found that use of the COVID-19 app did not save nor cost time, 29% (20/68) considered use saved time and 7% (5/68) said it cost them time (Table 5).

**Table 5:** COVID-19 app use (N = 71).

How many times have you used the COVID-19 app, n (%)		Value
	< 5 times	37 (52)
	5–10 times	26 (37)
	10–20 times	5 (7)
	>20 times	2 (3)
	Not applicable	1 (1)
**How long did it take to master the COVID-19 app, n (%)**		
	< = 3 times of use	57 (84)
	4–6 times of use	8 (12)
	> 6 times of use	3 (4)
**Did you use the app intuitively, n (%)**		
	Yes, I got it when I wanted to use it	19 (28)
	I sometimes thought of it to use the app to look up advice	27 (40)
	I never thought of using the app to look up advice	21 (31)
	At first I had to think of it, after a while it went automatically	1 (1)
**For what purpose did you use the app, n (%)**		
	Latest updates	47 (69)
	PPE instructions	11 (16)
	Infection prevention policy	36 (53)
	Clinical policy	16 (24)
	Look up / dial phone numbers	23 (34)
	Other	10 (15)
**In what situation did you use the app, n (%)**		
	In meetings to look up advice	7 (10)
	When walking from A to B	11 (16)
	When no computer available	15 (22)
	Slow computer	2 (3)
	To look up / dial phone numbers	15 (22)
	To quickly check something	43 (63)
	Other	7 (10)
**Influence on your work, n (%)**		
	App use saved me time	20 (29)
	App use neither saved me nor cost me time	43 (63)
	App use cost me time	5 (7)

Eighty-seven percent (62/71) of all questionnaire respondents thought that app use was moderately or fully culturally accepted in their work environment. However, none of the nurses in age group 20 to 29 (0/5) felt app use was fully accepted compared to 76% (13/17) of nurses in the other age groups. Age group was associated with the perception that app use was culturally accepted in the work environment (p<0.01). For example, most respondents in the age group 20–29 felt this was moderately accepted, while the majority of respondents of all other age groups felt it was fully accepted (S4 Appendix). Additionally, gender was associated with the perception that app use was culturally accepted in the work environment (p<0.01). None of the female respondents felt it was not accepted. Contrarily, male respondents did feel this way (S3 Appendix). There was a relationship between role and whether respondents would use the COVID-19 app adjacent to patients (p<0.01). None of the residents found this unprofessional, although medical specialists did (S4 Appendix). App use adjacent to patients was no problem for half of the respondents (36/68; 53%), a quarter had doubts to do so (19/68; 28%) while others, predominantly nurses (7/8), felt it was not right (8/68; 12%) and some thought it was unprofessional (5/68; 7%). Colleagues had no negative influence on app use and most respondents would (38/68; 56%) or might (27/68; 40%) recommend the COVID-19 app to their peers. Only 3% (2/68) of the respondents would not use a similar app with information and guidelines if it was to be introduced in our hospital in the future.

## Discussion

Here, we demonstrated that a smartphone app can be a very useful tool to disseminate up-to-date information and hospital policy to HCWs during a healthcare crisis such as the COVID-19 pandemic. Our COVID-19 app was implemented in a short period of time, was adopted easily by HCWs and cost little. Over the course of a 3⅓ month period, even after the peak of admitted patients, the app had 1168 users and 3903 unique sessions. The app was primarily used to access hospital policy regarding information for HCWs, such as when to test for SARS-CoV-2, latest updates, the SARS-CoV-2 / COVID-19 guideline and the COVID-19 telephone list. Considering the novelty of the virus and disease and changing insights it is not surprising information for HCWs regarding when to test for SARS-CoV-2 was accessed the most. Respondents of the questionnaire reflected the female:male ratio (75:25) of HCWs in our hospital (72:28). Respondents were positive about the influence apps had on their work and experienced an overall high user satisfaction using the COVID-19 app. One of the advantages was that the app can be used to quickly check information, while ‘on the go’ or in meetings, and predominantly had no effect on time or saved the user time. Additionally, previous non-users of apps seem to have a positive attitude towards this new technology for their work and are willing to adapt apps like the COVID-19 app.

Three months after its launch our app was downloaded by 1168 users, which is more than most other studies have reported for apps containing hospital policy. The dynamic work situation during the COVID-19 outbreak could explain the relatively high number of downloads of our app. The number of total app downloads in other studies varied from 53 times after 3 months, 1233 times in 4 months, 2013 times after 10 months, 990 times after 12 months to 3056 times after 14 months [19, 23, 25, 27, 28]. However, total app sessions (3903) seemed below average compared to other studies which ranged from 9259 sessions over 14 months, >16,000 sessions over 10 months to 18,860 sessions over 12 months and could be due to the short study period and novelty of the tool within the hospital [25, 28, 29]. Additionally, the app was introduced after the peak in COVID-19 admissions in the hospital and showed a corresponding decline in monthly use, similar to another study [16]. One study that was conducted during the start of the pandemic saw an increase in users, whereas our study conducted after the peak saw a decrease in COVID-19 app users, similar to the decrease in accessing COVID-19 related content in another study [18, 19]. We did not consider or intend the COVID-19 app to replace communication of the hospital policy via the intranet. However, since smartphones and apps are used extensively we aimed to augment the main channel of communication to increase the chance that SARS-CoV-2 / COVID-19 hospital policy reached HCWs. An article on the COVID-19 app was published on the intranet. Similar to survey respondents of our COVID-19 app, other studies found HCWs experienced their studied app as positive, easy and useful [17, 18].

Due to the very frequent updates in the COVID-19 app content we decided not to send push notifications to inform users of an update. This may have been a missed opportunity to engage more users as shown in another study [19]. Adherence to the COVID-19 content information in the app as well as the impact of the app on outcomes in patient care remain unclear and could be the focus of future research [18, 19].

Our COVID-19 app was used by all kind of HCWs. Indeed, hospitals are not only staffed with clinical workers such as physicians and nurses but also with other HCWs including technicians, administrative professionals and research and education professionals. During the COVID-19 crisis extra effort was asked of every HCW. Many HCWs got their COVID-19 information, such as risk of acquiring and transmitting SARS-CoV-2, from social media which may lead to poorer understanding or misunderstanding on several topics [30, 31]. Additionally, unclear and frequently changing guidelines can cause confusion amongst HCWs [32]. Therefore, hospital management needs to ensure that all HCWs receive accurate and up-to-date information in a timely fashion. In this technological era a smartphone app is an accessible, easy-to-use and an universal tool to facilitate dissemination of information.

Remarkably, none of the nurses in the youngest age category felt app use was fully accepted in their work environment contrary to the older age groups. This may be associated with a junior position of these HCWs who might worry that others perceive their smartphone use as not work-related [33]. Age group and gender were associated with the perception that app use was culturally accepted in the work environment. Additionally, a new group of app users who previously did not use apps for their work were enthusiastic to use our app during the COVID-19 crisis (13/71; 18%). Most would use or consider use of a similar future app which illustrates the willingness to adapt a new way of working in order to combat a new, not fully understood virus and disease.

Most questionnaire respondents felt app use for work was somewhat (12/71; 17%) or fully (50/71; 70%) accepted in their work environment and did not think it would be a problem to use the app adjacent to patients if necessary. Role was associated whether respondents would use the COVID-19 app adjacent to patients. Seven percent (5/68) of the respondents considered app use adjacent to patients unprofessional and 12% (8/68) stated that it did not feel right. Although we do not know the underlying reasons for this, as this was not explored, there are several possibilities. Mobile phones are known to be contaminated with a variety of pathogens [34]. Mobile phones in the hospital should therefore be used with application of basic infection prevention measures, but this may not be known by HCWs which might have made them reluctant to use the app. Additionally, HCWs might think they give the impression using their phone for leisure in front of the patient. Similarly, other studies showed that HCWs may feel uncomfortable (20%) using an app near colleagues or patients [23, 33]. Increasing awareness of the use of smartphone apps as medical tools remains necessary to allow them reaching their full potential in healthcare institutions.

### Strengths and limitations

A strength of this study was that we evaluated the use of a relatively novel tool in a hospital during a healthcare crisis, which has been studied very limited. A possible limitation is the short period of this study which started after the peak and during the decline of COVID-19 in our hospital, and this has to be weighed against the corresponding onboarding, plateau phase and decline in app use. HCWs also may have been already familiarized with hospital’s intranet as the go-to source of SARS-CoV-2 / COVID-19 information at the start of the study. Data on how often hyperlinks to source documents were clicked were not available, and we could not determine if app users had any interest in the hyperlinks. Additionally, the questionnaire used in this pilot study was based on preliminary data from an ongoing study. A limited number of app users from all categories of HCWs participated in the questionnaire and users with a positive experience might be more inclined to do so, leading to a bias in appreciation of the app.

## Conclusions

In a healthcare crisis HCWs need to be able to access an up-to-date hospital policy at all times in order to fulfill their jobs effectively and correctly. Nowadays, most HCWs carry a smartphone providing an ideal medium to receive critical information through apps during a pandemic such as COVID-19. Short implementation time makes it an excellent hospital resource to have in dire times. Our COVID-19 app proved to be a relatively simple yet innovative tool that was used by HCWs from all disciplines involved in care of COVID-19 patients. The up-to-date app was used for different topics and had a high user satisfaction amongst questionnaire respondents. The short implementation time makes it an attractive tool to disseminate information on topics such as infection prevention and control, use of PPE and patient care. An app with local hospital policy can be a invaluable tool during a pandemic.

## Supporting information

S1 Fig COVID-19 app use by time of day. (TIF)

S1 Appendix Questionnaire in English and Dutch. (DOCX)

S2 Appendix User analytics–number of users per role. (DOCX)

S3 Appendix User analytics—top most frequently accessed advice. (DOCX)

S4 Appendix Association between demography and survey responses. (DOCX)
