# Supplementary figures and images for: Use of a smartphone app to inform healthcare workers of hospital policy during a pandemic such as COVID-19: A mixed methods observational study
Source: PLoS One. 2022 Jan 5;17(1):e0262105. doi: 10.1371/journal.pone.0262105 (PMC8730417; doi:10.1371/journal.pone.0262105)

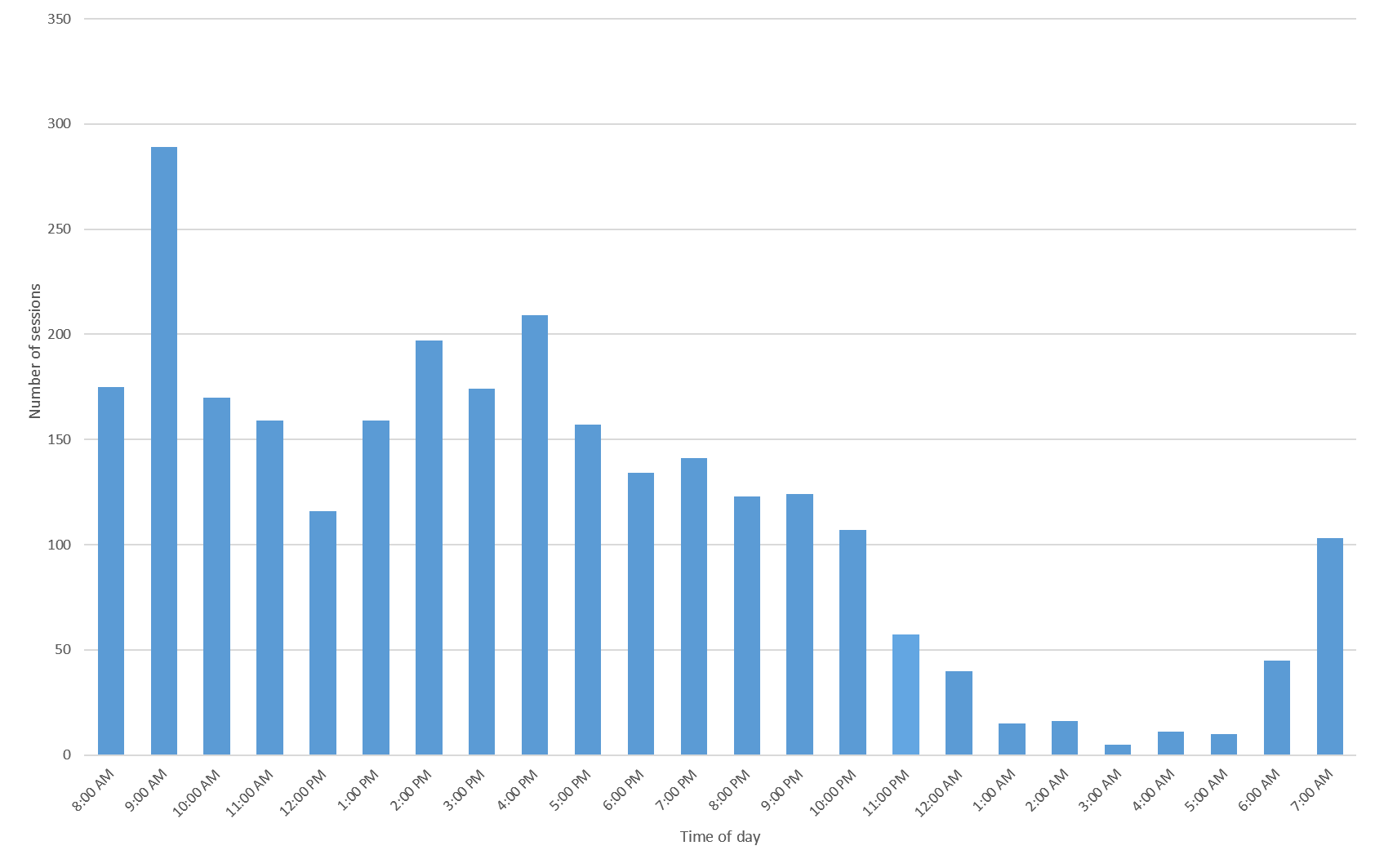

Supplement: S1 Fig — (TIF) [file pone.0262105.s001.tif]
