# Supplementary material for: Use of a smartphone app to inform healthcare workers of hospital policy during a pandemic such as COVID-19: A mixed methods observational study
Source: PLoS One. 2022 Jan 5;17(1):e0262105. doi: 10.1371/journal.pone.0262105 (PMC8730417; doi:10.1371/journal.pone.0262105)
Supplement: S1 Appendix — (DOCX) [file pone.0262105.s002.docx]

COVID-19 app questionnaire (English)

General

1. What is your age?
2. What is your gender?
3. What is your role in the hospital?
   1. Resident
   2. Medical specialist
   3. Nurse
   4. Medical student
   5. Fellow
   6. Infection preventionist
   7. Management
   8. Physician assistant
   9. Other
4. For which department do you work (normally)?
5. How do you feel about smartphone app use for work?
   1. Apps can contribute to my work
   2. Apps don’t contribute to my work
   3. No opinion
6. Is the use of smartphone apps (culturally) accepted in your work environment?
   1. Yes
   2. Moderately
   3. No
   4. I don’t know
7. Do you ever user smartphone apps other than the COVID-19 app for work?
   1. Yes, daily
   2. Yes, a few times a week
   3. Yes, a few times a month
   4. No
8. Do you use smartphone apps for work on a personal smartphone or work smartphone?
   1. Personal smartphone
   2. Work smartphone
   3. Both
   4. Not applicable

COVID-19 app

1. What were your first impressions of the COVID-19 app?
   Select what applies:

- Accessible / inaccessible
- Fast / Slow
- Clear / unclear
- Complete / incomplete
- Easy to use / difficult to use
- Useful / Not useful

1. Do you trust the COVID-19 app?
   1. Yes
   2. After comparing the content to the content of the original source I did
   3. A little
   4. No
   5. Not applicable
2. How many times have you used the COVID-19 app in total?
   1. <5 times
   2. 5 – 10 times
   3. 10 – 20 times
   4. >20 times
   5. Not applicable
3. How intuitive was it to use the COVID-19?
   1. Intuitive. I used the COVID-19 app when I wanted to look up something
   2. A little intuitive. I sometimes thought of using the COVID-19 app to look up something
   3. Not intuitive. I never thought of using the COVID-19 when I wanted to look up something
   4. At first I had to think of using the COVID-19 app. After some time I opened the app automatically to look up something
   5. Not applicable
4. How long did it take to get to know the COVID-19?
   1. After 3 times of use or less
   2. After 4 to 6 times of use
   3. After more than 6 times of use
   4. Not applicable
5. Were you familiar with the advice in the COVID-19 app?
   1. Yes
   2. With a part of the advice I was familiar
   3. No, the advice was new for me
   4. Not applicable
6. For what purpose did you use the COVID-19 app generally?

- To stay up to date on SARS-CoV-2 / COVID-19 advice
- Instructions for PPE
- Infection prevention policy
- Clinical policy
- Looking up / calling phone numbers
- Other
- Not applicable

1. In what situation did you use the COVID-19 app?

- In meetings if looking up something was necessary
- When I walked through the corridors and had to look up something
- If there was no computer available
- If the computer was slow
- If I wanted to look up / call and phone number
- If I wanted to check something quickly
- Not applicable

1. In some of the advice in the COVID-19 app a link was used to guide you to information on a website. How did you feel about that?
   1. That was no problem
   2. That was annoying
   3. I didn’t encounter that
   4. Not applicable
2. Influence of the COVID-19 app on your work
   1. App use saved me time
   2. App use cost me time
   3. App use neither saved nor cost me time
   4. Not applicable
3. What kind of influence did colleagues have on your COVID-19 app use?
   1. Motivating
   2. Demotivating
   3. No influence
   4. Not applicable
4. Would you recommend the COVID-19 app to colleagues?
   1. Yes
   2. No
   3. Maybe
   4. Not applicable
5. Would you use the COVID-19 app adjacent to patients?
   1. If it’s necessary I would not have a problem with it
   2. No, I think it is unprofessional
   3. No, that doesn’t feel right
   4. I don’t know
   5. Not applicable
6. If this hospital would introduce a similar app with advice and guidelines in the future, would you use it?
   1. If such an app would be introduced I would use it
   2. No, I would not use such an app
   3. Maybe I would use such an app

No answer

1. Do you have any feedback, tips or thoughts on the COVID-19 app you’d like to share?

<free text>

COVID-19 app questionnaire (Dutch)

Algemeen

1. Wat is uw leeftijd?
2. Wat is uw geslacht?
   1. Man/vrouw aanklikken
3. Wat is uw rol in het ziekenhuis?
   1. Arts in opleiding tot specialist (AIOS)
   2. Arts niet in opleiding tot specialist (ANIOS)
   3. Arts-onderzoeker
   4. Coassistent / Geneeskundestudent
   5. Fellow
   6. Infectiepreventie deskundige
   7. Management
   8. Medisch specialist
   9. Physician assistant / nurse practioner
   10. Verpleegkundige
   11. Overig
4. Op welke afdeling werkt u (normaal gesproken)?
5. Hoe denkt u over het gebruik van smartphone apps voor werk?
   1. Apps kunnen bijdragen aan mijn werkzaamheden
   2. Apps dragen niks bij aan mijn werkzaamheden
   3. Geen mening
6. Is het gebruik van smartphone apps voor uw werk (cultureel) geaccepteerd in uw werkomgeving?
   1. Ja
   2. Enigszins
   3. Nee
   4. Weet ik niet
7. Maakt u wel eens gebruik van smartphone apps (anders dan de COVID-19 app) voor uw werk?
   1. Ja, elke dag
   2. Ja, een paar keer per week
   3. Ja, een paar keer per maand
   4. Nee
8. Gebruikt u smartphone apps voor werk op uw privételefoon of op een werktelefoon?
   1. Privételefoon
   2. Werktelefoon
   3. Beide
   4. Niet van toepassing

COVID-19 app

1. Wat waren uw eerste indrukken van de COVID-19 app?

- Overzichtelijk / onoverzichtelijk
- Snel / traag
- Duidelijk / onduidelijk
- Compleet / incompleet
- Makkelijk in gebruik / moeilijk in gebruik
- Nuttig / overbodig

1. Heeft u vertrouwen in de COVID-19 app?
   1. Ja
   2. Na vergelijking met de adviezen op Agora op juistheid had ik vertrouwen
   3. Een beetje
   4. Nee
   5. Niet van toepassing
2. Hoe vaak heeft u in totaal gebruik gemaakt van de COVID-19 app?
   1. <5 keer
   2. 5 – 10 keer
   3. 10 – 20 keer
   4. >20 keer
   5. Niet van toepassing
3. Hoe intuïtief was het om de COVID-19 app te gebruiken?
   1. Intuïtief, ik pakte de COVID-19 app erbij als ik een advies wilde opzoeken
   2. Een beetje intuïtief, ik dacht er soms aan om de COVID-19 app erbij te pakken om een advies op te zoeken
   3. Niet intuïtief, ik dacht er eigenlijk nooit aan om de COVID-19 app erbij te pakken om een advies op te zoeken
   4. In het begin moest ik er aan denken om de COVID-19 app te gebruiken, later ging dat vanzelf
   5. Niet van toepassing
4. Hoe lang duurde het voordat u gebruik van de COVID-19 app onder de knie had?
   1. Na 3 keer of minder gebruiken
   2. Na 4 tot 6 keer gebruiken
   3. Na meer dan 6 keer gebruiken
   4. Niet van toepassing
5. Was u reeds bekend met de adviezen in de COVID-19 app?
   1. Ja
   2. Met een deel van de adviezen was ik bekend
   3. Nee, het waren nieuwe adviezen voor mij
   4. Niet van toepassing
6. Waarvoor heeft u de COVID-19 app vooral gebruikt?

- Up to date blijven van laatste nieuws omtrent SARS-CoV-2 / COVID-19 adviezen
- Instructies voor gebruik PPE
- Infectiepreventie beleid
- Klinisch beleid
- Telefoonnummers opzoeken/bellen
- Anders
- Niet van toepassing

1. In welke situatie gebruikte u de COVID-19 app?

- In vergaderingen/overdrachten als het opzoeken van een advies nodig was
- Als ik van A naar B liep en iets op wilde zoeken
- Als er geen computer beschikbaar was
- Als de computer traag was
- Als ik een telefoonnummer wilde opzoeken/bellen
- Als ik iets snel wilde nakijken
- Niet van toepassing

1. Bij een deel van de adviezen in de COVID-19 app stond een link naar het document op Agora. Wat vond u hiervan?
   1. Dit vond ik geen probleem
   2. Dit vond ik storend
   3. Die adviezen ben ik niet tegengekomen
   4. Niet van toepassing
2. Invloed van de COVID-19 app op uw werkzaamheden
   1. Gebruik van de app leverde tijdswinst op
   2. Gebruik van de app kostte tijd
   3. Gebruik van de app leverde geen tijdswinst op en kostte ook geen tijd
   4. Niet van toepassing
3. Wat voor invloed hadden collega’s om de COVID-19 app te gebruiken?
   1. Motiverend
   2. Demotiverend
   3. Geen invloed
   4. Niet van toepassing
4. Zou u de COVID-19 app aan collega’s adviseren?
   1. Ja
   2. Nee
   3. Misschien
   4. Niet van toepassing
5. Zou u de COVID-19 app in het bijzijn van patiënten gebruiken?
   1. Als dit nodig is, zou ik het geen probleem vinden
   2. Nee, dit vind ik onprofessioneel
   3. Nee, dat voelt niet prettig
   4. Weet ik niet
   5. Niet van toepassing
6. Zou u in de toekomst een vergelijkbare app met adviezen en richtlijnen van het Erasmus MC gebruiken?
   1. Als zo’n app zou worden geïntroduceerd zou ik die gebruiken
   2. Nee, ik zou zo’n app niet gebruiken
   3. Wellicht zou ik zo’n app gebruiken
7. Heeft u nog op- of aanmerkingen of tips?

<vrij tekstveld>
