# Supplementary material for: Use of a smartphone app to inform healthcare workers of hospital policy during a pandemic such as COVID-19: A mixed methods observational study
Source: PLoS One. 2022 Jan 5;17(1):e0262105. doi: 10.1371/journal.pone.0262105 (PMC8730417; doi:10.1371/journal.pone.0262105)
Supplement: S2 Appendix — (DOCX) [file pone.0262105.s003.docx]

| **Role** | **Number of users, n (%)** |
| --- | --- |
| Medical specialist | 261 (22.3) |
| Nurse | 259 (22.2) |
| Resident | 193 (16.5) |
| Management | 72 (6.2) |
| Medical student | 47 (4.0) |
| Fellow | 25 (2.1) |
| Infection prevention expert | 5 (0.4) |
| Other* | 306 (26.2) |
