# Supplementary material for: Use of a smartphone app to inform healthcare workers of hospital policy during a pandemic such as COVID-19: A mixed methods observational study
Source: PLoS One. 2022 Jan 5;17(1):e0262105. doi: 10.1371/journal.pone.0262105 (PMC8730417; doi:10.1371/journal.pone.0262105)
Supplement: S3 Appendix — (DOCX) [file pone.0262105.s004.docx]

| **Top most frequently accessed advice** | **Number of accessions** |
| --- | --- |
| Information for HCWs such as when to test for SARS-CoV-2 | 1214 |
| Latest updates | 1181 |
| COVID-19 telephone list | 418 |
| SARS-CoV-2 / COVID-19 guideline | 280 |
| Information regarding patient care | 252 |
| SARS-CoV-2 / COVID-19 therapy | 185 |
| Information regarding diagnostics and isolation | 182 |
| Frequently asked questions | 151 |
| Letter templates to COVID-19 patients | 141 |
| Instructions on personal protective equipment (PPE) | 115 |
| **Top most frequently accessed screens** |  |
| HCWs with possible COVID-19 symptoms | 596 |
| No / unknown unprotected physical contact with a COVID-19 patient | 472 |
| HCW without fever | 461 |
| Contact tracing of HCWs | 268 |
| Frequently asked questions | 267 |
| Physical contact with patients | 180 |
| Physical contact without adequate PPE | 162 |
| HCW with >=1 clinical symptoms | 157 |
| HCW with fever | 155 |
| When to end isolation | 117 |
