# Supplementary material for: Use of a smartphone app to inform healthcare workers of hospital policy during a pandemic such as COVID-19: A mixed methods observational study
Source: PLoS One. 2022 Jan 5;17(1):e0262105. doi: 10.1371/journal.pone.0262105 (PMC8730417; doi:10.1371/journal.pone.0262105)
Supplement: S4 Appendix — (DOCX) [file pone.0262105.s005.docx]

|  | | **Is use of smartphone apps for your work culturally accepted in your work environment?, n (%)** | | | | |
| --- | --- | --- | --- | --- | --- | --- |
|  |  | Moderately | Yes | No | I don't know | Total |
| Age category | 20 - 29 | 7 (70.0) | 2 (20.0) | 0 (0.0) | 1 (10.0) | 10 (100) |
|  | 30 - 39 | 0 (0.0) | 8 (88.9) | 0 (0.0) | 1 (11.1) | 9 (100) |
|  | 40 - 49 | 2 (11.1) | 12 (66.7) | 0 (0.0) | 4 (22.2) | 18 (100) |
|  | 50 - 59 | 1 (3.6) | 25 (89.3) | 1 (3.6) | 1 (3.6) | 28 (100) |
|  | 60 - 69 | 2 (33.3) | 3 (50.0) | 1 (16.7) | 0 (0.0) | 6 (100) |
| Total | | 12 (16.9) | 50 (70.4) | 2 (2.8) | 7 (9.9) | 71 (100) |

|  | | **Is use of smartphone apps for your work culturally accepted in your work environment?, n (%)** | | | | |
| --- | --- | --- | --- | --- | --- | --- |
|  |  | Moderately | Yes | No | I don't know | Total |
| Gender | Male | 6 (33.3) | 10 (55.6) | 2 (11.1) | 0 (0.0) | 18 (100) |
|  | Female | 6 (11.3) | 40 (75.5) | 0 (0.0) | 7 (13.2) | 53 (100) |
| Total | | 12 (16.9) | 50 (70.4) | 2 (2.8) | 7 (9.9) | 71 (100) |

|  | | **Would you use the COVID-19 app adjacent to patients, n (%)** | | | | |
| --- | --- | --- | --- | --- | --- | --- |
|  |  | If it was necessary, I would not have a problem with it | No, it does not feel right | No, I think it is unprofessional | I don't know | Total |
| Role in the hospital | Resident | 3 (75.0) | 0 (0.0) | 0 (0.0) | 1 (25.0) | 4 (100) |
|  | Management | 7 (70.0) | 0 (0.0) | 0 (0.0) | 3 (30.0) | 10 (100) |
|  | Medical specialist | 7 (63.6) | 0 (0.0) | 2 (18.2) | 2 (18.2) | 11 (100) |
|  | Other | 4 (22.2) | 1 (5.6) | 3 (16.7) | 10 (55.6) | 18 (100) |
|  | Physician assistant / nurse practitioner | 3 (100) | 0 (0.0) | 0 (0.0) | 0 (0.0) | 3 (100) |
|  | Nurse | 12 (54.5) | 7 (31.8) | 0 (0.0) | 3 (13.6) | 22 (100) |
| Total | | 36 (52.9) | 8 (11.8) | 5 (7.4) | 19 (27.9) | 68 (100) |
